# The Role of Direct Oral Anticoagulants (DOACs) in Cancer-Associated Thrombosis: A Comprehensive Review of the Literature

**DOI:** 10.7759/cureus.83956

**Published:** 2025-05-12

**Authors:** Muzammil R Qureshi, Marian Barsoum, Danyal Salim, Sese Ekolle Mbonde Mudika, Aly Barakat, Neeraja Reghu Nair, Tanzeela F Ahmed, Shirish Nayyar, Aya H Dahleh, Sidra Ambreen, Aamanda Noel, Muteeba Fayyaz

**Affiliations:** 1 Internal Medicine, Avalon University School of Medicine, Willemstad, CUW; 2 Graduate Medical Education, Faculty of Medicine, Assiut University, Assiut, EGY; 3 Internal Medicine, "Grigore T. Popa" University of Medicine and Pharmacy, Lasi, ROU; 4 General Medicine, University of Yaoundé 1, Yaoundé, CMR; 5 Internal Medicine, Medway Maritime Hospital NHS Foundation Trust, Gillingham, GBR; 6 General Medicine, Shridevi Institute of Medical Sciences and Research Hospital, Tumkur, IND; 7 General Medicine, Shree Haridev Joshi General Hospital, Dungarpur, IND; 8 Internal Medicine, King’s College Hospital, London, GBR; 9 Internal Medicine, Royal College of Surgeons in Ireland, Dublin, IRL; 10 Medical Education, The University of Texas Health Science Center at Houston, Houston, USA; 11 Emergency Medicine, Arima General Hospital, Arima, TTO; 12 Emergency Medicine, Norfolk and Norwich University Hospital NHS Foundation Trust, Norwich, GBR

**Keywords:** anticoagulation in cancer, apixaban, bleeding risk in malignancy, cancer-associated thrombosis (cat), chemotherapy-induced thrombosis, direct oral anticoagulants (doacs), low-molecular-weight heparin (lmwh), rivaroxaban, thromboprophylaxis, venous thromboembolism (vte)

## Abstract

Venous thromboembolism (VTE) is a frequent and serious complication observed in patients with malignancy. The management of cancer-associated thrombosis (CAT) remains complex and multifactorial, influenced by cancer type, stage, comorbidities, and ongoing therapeutic regimens. Among anticoagulation strategies, direct oral anticoagulants (DOACs) have gained increasing attention as potential alternatives to low-molecular-weight heparins (LMWHs) in selected oncology populations. This narrative review evaluates the role of DOACs in the treatment of CAT, focusing on their clinical efficacy, safety considerations, and practical advantages, including oral administration and patient adherence. However, their use may be limited in certain cancer types and patients with renal or hepatic impairment. DOACs have also raised concerns regarding bleeding risks, drug interactions, and individualized dosing strategies. This review also highlights ongoing challenges such as optimal treatment duration, real-world applicability, and patient-specific considerations. The discussion aims to assess current data and clinical guidance while identifying future directions for integrating DOACs into standard oncology practice.

## Introduction and background

**Figure 1:**
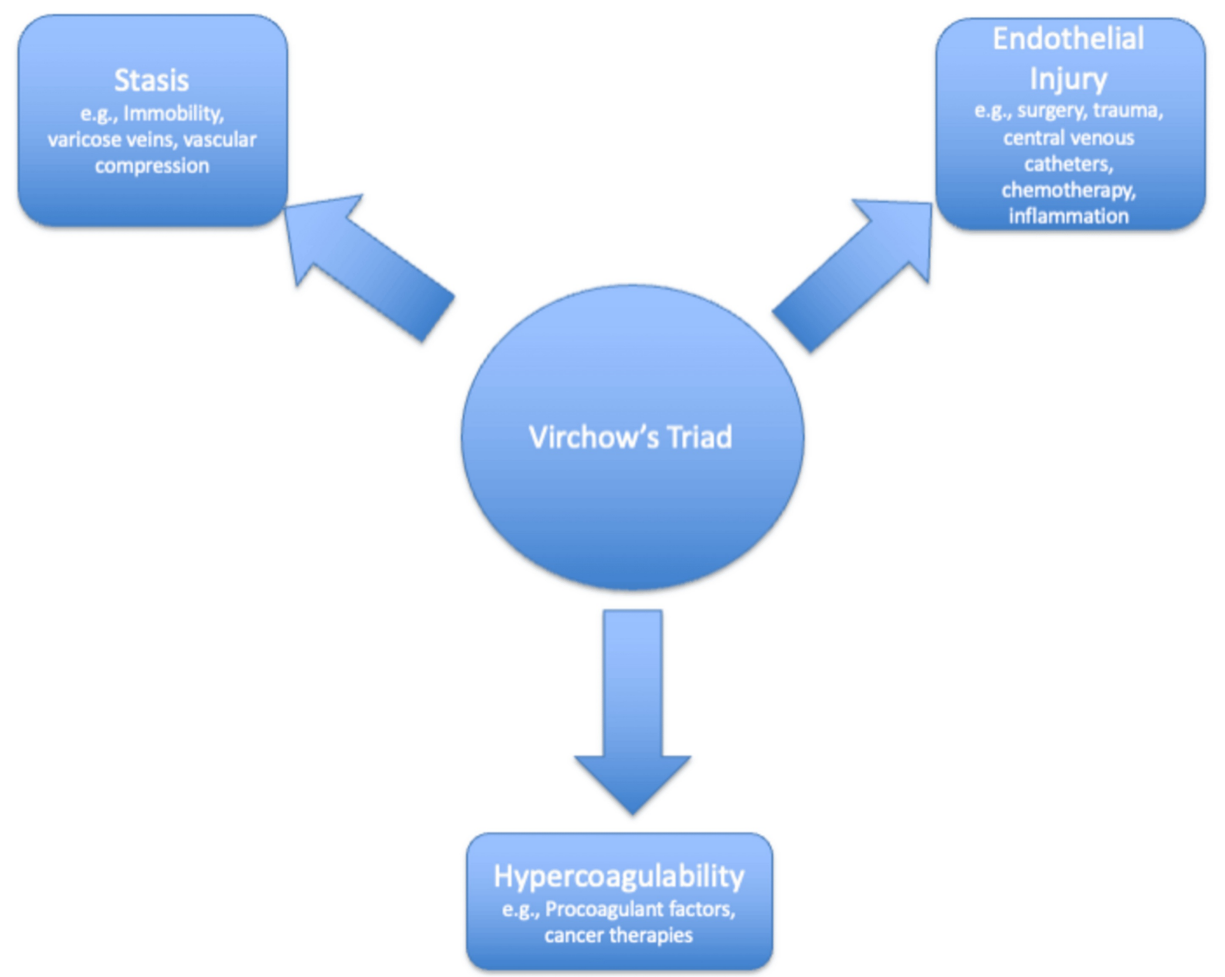
Virchow's triad in cancer patients Image Credit: Authors' original creation. This image was created using PowerPoint (Microsoft Corp., Redmond, WA, US).

Venous thromboembolism (VTE) is a critical condition characterized by the formation of blood clots in the veins, encompassing deep vein thrombosis (DVT) and pulmonary embolism (PE) within its clinical spectrum [1]. In oncology, VTE is a prevalent condition, as it ranks the second leading cause of mortality among cancer patients [2]. Approximately 20% of cases occur in individuals with underlying malignancies, especially during the active perioperative phase. Cancer patients exhibit a four- to sevenfold increased likelihood of developing VTE compared to the general population, along with an elevated risk of recurrence [3]. Virchow’s triad drives the heightened vulnerability: endothelial damage induced by several factors such as chemotherapy, stasis due to factors like immobility, and hypercoagulability resulting from elevated procoagulant factors and inflammatory markers (Figure 1) [4]. Cancer-specific factors, such as type, stage, associated comorbidities, and treatment regimens, significantly influence the risk of the disease. Hematologic malignancies, pancreatic cancer, and brain tumors are notably linked to an increased incidence of VTE [5]. Managing cancer-associated thrombosis (CAT) poses a complex challenge. The complexity of treatment is influenced by the type of cancer, coexisting medical conditions, disease stage, and therapy-related factors. Recent guidelines from the American Society of Clinical Oncology (ASCO) and the International Society on Thrombosis and Hemostasis (ISTH) recommend the use of direct oral anticoagulants (DOACs) instead of low-molecular-weight heparin (LMWH) for specific patient subsets with CAT. DOACs, such as apixaban and rivaroxaban, exhibit similar efficacy and safety profiles to LMWH while providing the notable benefit of oral administration, thereby improving patient adherence and facilitating long-term treatment. Nonetheless, their application presents certain limitations. Patients diagnosed with upper gastrointestinal cancers or other malignancies that present elevated bleeding risks necessitate a careful and tailored management strategy [6]. Considerations include renal impairment, extreme body weight, thrombocytopenia, and concurrent use of angiogenesis inhibitors or other therapies that may increase bleeding or recurrent VTE risks. In this review article, we aim to provide a comprehensive overview of the role of DOACs in managing CAT and evaluate their efficacy, safety, and comparative effectiveness with other anticoagulant therapies. 

DOACs, such as apixaban, rivaroxaban, edoxaban, and dabigatran, are increasingly being used in the treatment of CAT [7]. In recent clinical practice, DOACs have emerged as the preferred option in preventing VTE due to their superiority or noninferiority to prior therapies like LMWHs and vitamin K antagonists (VKAs) [8]. Patients with cancer have been shown to have a poor tolerance to daily injections for extended periods, which results in poor compliance and increased recurrence rates [9]. DOACs are administered orally, have a rapid onset of action and more predictable pharmacodynamics than VKAs, do not require routine laboratory monitoring of anticoagulant effect, present a wider therapeutic index, have more rapid onset of action, and have short half-lives. In terms of safety, DOACs are associated with a significantly lower risk of intracranial hemorrhage compared to VKAs [10]. Their overall bleeding profile is also more favorable [11], and they are not associated with serious adverse events such as warfarin-induced skin necrosis or calciphylaxis [12]. Additionally, DOACs demonstrate fewer drug and food interactions. It is also notable to mention that VKAs could be affected by the diet regime the patient is on [13]. This reduced interaction profile contributes to improved safety, ease of use, and patient adherence in clinical settings.

DOACs are categorized into two main classes: oral direct factor Xa inhibitors (e.g., rivaroxaban, apixaban, and edoxaban) and direct thrombin inhibitors (such as dabigatran). Factor Xa inhibitors work directly and reversibly by inhibiting factor Xa in the coagulation cascade. Dabigatran, on the other hand, selectively and reversibly inhibits factor IIa (thrombin) in both thrombus-bound and free forms.

CAT significantly increases morbidity, mortality, and healthcare costs in patients with malignancy. The management of VTE in cancer patients is especially challenging due to elevated risks of both thrombosis recurrence and bleeding complications during anticoagulation therapy. Compared to non-cancer VTEs, these patients experience approximately twice the recurrence rate and triple the risk of major bleeding [14]. Current clinical guidelines recommend DOACs as primary prophylaxis in ambulatory cancer patients at intermediate or high risk of thrombosis who are receiving systemic therapy, particularly apixaban or rivaroxaban. For initial treatment of active cancer with acute VTE, apixaban is often preferred over rivaroxaban due to its more favorable bleeding profile during the first week of therapy. The typical anticoagulation duration ranges from three to six months and is often extended as long as the cancer remains active [15].

## Review

Methodology

A comprehensive literature search was performed using PubMed to identify studies published between 2008 and 2025. The search strategy included a combination of Medical Subject Headings (MeSH) and free-text terms related to cancer (e.g., neoplasm, malignancy), VTE (e.g., CAT), and DOACs (e.g., LMWH, VKAs).

Filters were applied to include only human studies published in English. Boolean operators (AND, OR) were used to refine results. Additionally, reference lists of included studies were screened to include any relevant studies missed in the initial search, and further key studies were incorporated when necessary to support the goal of this review.

A total of 557 studies were initially identified from the database. After applying filters to include only clinical trials, meta-analyses, and randomized controlled trials, 130 studies remained. At this stage, 427 studies were excluded due to one or more of the following: editorials, commentaries, biographies, bibliographic entries, case reports, non-English language, and not focused on CAT.

From the 130 filtered studies, only 69 had free full-text access and were retained for further screening, excluding 61 studies. When screening for the titles and abstracts, 31 of the 69 studies were then excluded for reasons including pediatric population, non-cancer thrombosis, and topics outside the scope of this review. This left a total of 38 studies that were included in the final review based on full eligibility and relevance.

This review included peer-reviewed original research articles, studies involving adult patients with CAT, randomized controlled trials, meta-analyses, and articles available in English with full-text access.

Editorials, commentaries, biographies, bibliographic items, case reports, non-English language or unavailable full-text access, studies not directly addressing the use of DOACs in CAT, and studies including pediatric-only populations were excluded.

Screening process

**Figure 2:**
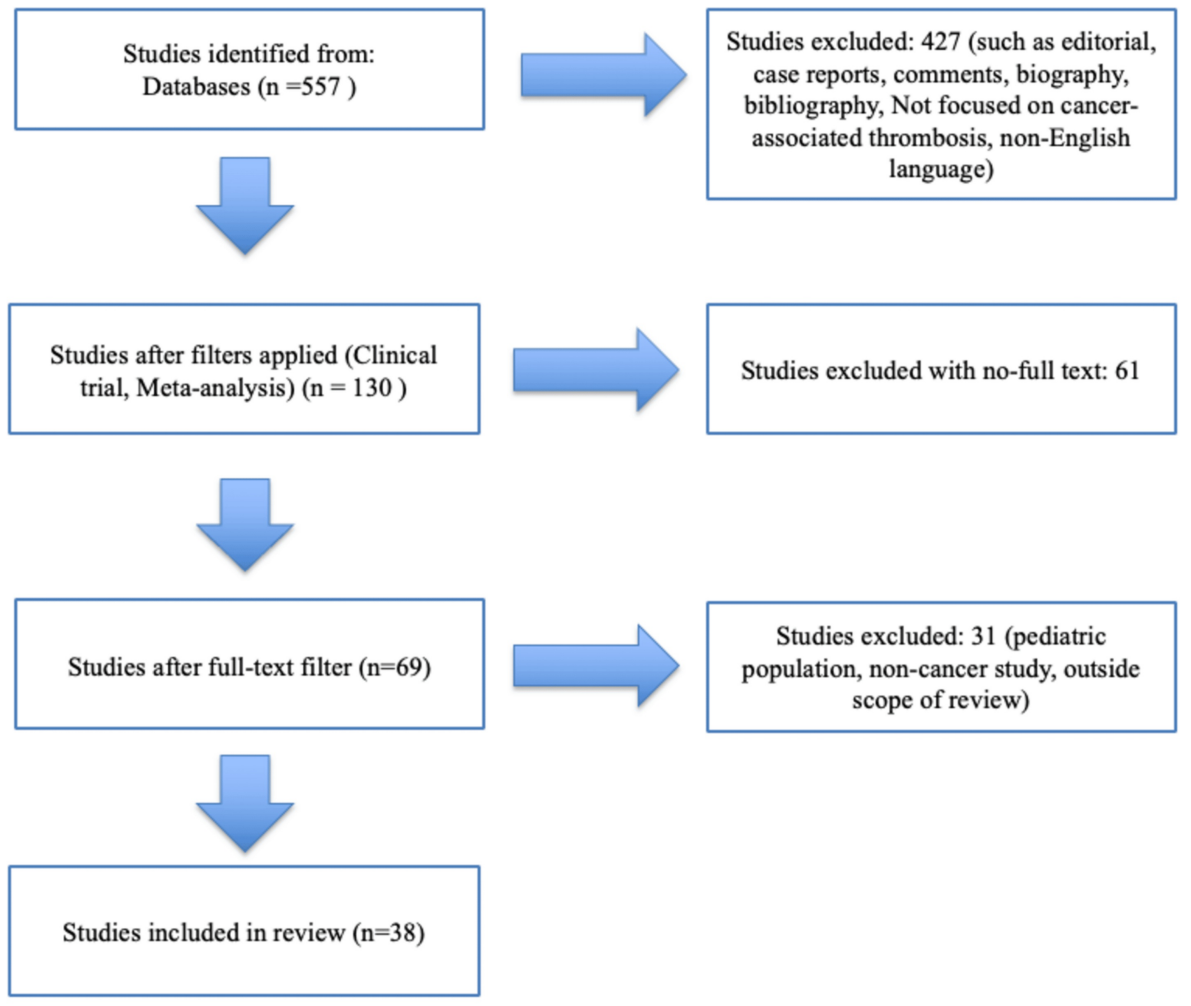
Stepwise flowchart of the screening, inclusion, and exclusion processes for eligible studies

All studies were screened manually by the authors based on title, abstract, and full-text review. Data extraction focused on study design, sample size, anticoagulants used, and clinical outcomes such as VTE recurrence and bleeding (Figure 2).

Comparative efficacy and safety of DOACs and LMWH in CAT

Advantages of DOACs in CAT

Recent advancements in the management of CAT have highlighted the growing role of DOACs, particularly in light of the emerging evidence supporting their efficacy and convenience. Clinical trials such as the Caravaggio [16], SELECT-D [17], and Hokusai VTE Cancer [9] trials have demonstrated that DOACs are at least noninferior to LMWH in preventing VTE recurrence in cancer patients. One of the major advantages of DOACs is their oral administration, which eliminates the need for daily subcutaneous injections and has been associated with better patient adherence and satisfaction, particularly in the outpatient setting. In addition, DOACs do not require routine laboratory monitoring, reducing healthcare burden and making them suitable for long-term anticoagulation. Their rapid onset of action, predictable pharmacokinetics, and fewer food-drug interactions compared to VKAs further support their growing clinical use. Recent studies have also explored reduced-dose regimens of agents like apixaban for secondary prevention, balancing efficacy with bleeding risk, and further improving the risk-benefit profile in selected patients [8]. For these reasons, major clinical guidelines (e.g., ASCO, ISTH) now support the use of DOACs as a potential first-line option for many patients with CAT.

Limitations and considerations in DOAC use

Despite their benefits, the use of DOACs in cancer patients requires careful individualized consideration, as not all patients are ideal candidates. Certain malignancies, particularly those involving the gastrointestinal or genitourinary tracts, have been associated with higher bleeding risk when treated with DOACs, especially rivaroxaban and edoxaban. Patients with advanced renal or hepatic impairment, extreme body weight, or thrombocytopenia present additional challenges, as pharmacokinetics and bleeding risks may be altered in these settings. In such populations, LMWH remains preferred due to its shorter half-life, established safety profile, and dosing flexibility [18,19]. Moreover, the limited representation of complex cancer cases in randomized trials raises questions about generalizability; most studies have been conducted in controlled environments, potentially underrepresenting patients with poor performance status or those on intensive chemotherapy. Additionally, drug-drug interactions with certain anticancer agents, such as CYP3A4 or P-glycoprotein inhibitors, may influence DOAC metabolism, further complicating their routine use in oncology. Many anticancer therapies, particularly tyrosine kinase inhibitors, hormonal therapies, and immune-modulating agents, interact with either P-gp or CYP3A4 (the two major metabolic pathways affecting DOAC pharmacokinetics, especially for rivaroxaban and apixaban). For example, inhibitors such as cyclosporine, tacrolimus, and imatinib may increase DOAC plasma concentrations, thereby elevating bleeding risk. In contrast, inducers like dexamethasone and vinblastine may lower DOAC levels, potentially reducing their anticoagulant efficacy and increasing thrombotic risk. Anthracyclines, commonly used in cancer treatment, are notable both as P-gp inducers (e.g., doxorubicin) and for their potential direct cardiotoxic effects, further complicating anticoagulation management [20]. As the long-term safety of DOACs in diverse cancer subtypes remains under investigation, treatment decisions should be guided by comprehensive risk assessment, multidisciplinary input, and ongoing monitoring of renal function, bleeding signs, and treatment response. A head-to-head comparison of DOACs and LMWH is outlined in Table 1.

**Table 1:** Comparisons between DOACs and LMWH DOACs: direct oral anticoagulants, LMWH: low-molecular-weight heparin, VTE: venous thromboembolism, GI: gastrointestinal, GU: genitourinary, VKAs: vitamin K antagonists.

Parameters	DOACs	LMWH
Administration	Oral administration	Subcutaneous injections
Efficacy	Comparable to LMWH for VTE treatment	Comparable to DOACs for VTE treatment
Safety	Requires dose adjustment in renal impairment; bleeding risk may increase in GI/GU cancers; some interactions with chemotherapy possible	Dose adjustment in renal impairment; bleeding risk exists but typically lower in GI/GU cancers; injectable route may be less preferred during chemotherapy
Adherence	Higher due to oral route and ease of use	Lower due to discomfort and burden of injections
Duration of therapy	Potential for longer-term use	Commonly used for 3-6 months
Drug interactions	Fewer than VKAs; CYP3A4/P-gp interactions possible; requires renal function monitoring	Fewer than VKAs; requires renal function monitoring
Monitoring requirement	No routine coagulation monitoring	No routine coagulation monitoring

Main clinical data evaluating DOACs in cancer patients

A meta-analysis of 2,607 patients comparing DOACs to LMWHs in CAT showed a nonsignificant reduction in recurrent VTE with DOACs (RR 0.68, 95% CI 0.39-1.17), alongside nonsignificant increases in major bleeding (RR 1.36, 95% CI 0.55-3.35) and clinically relevant non‐major bleeding (CRNMB; RR 1.63, 95% CI 0.73-3.64). The composite outcome of recurrent VTE or major bleeding (RR 0.86, 95% CI 0.60-1.23) and all-cause mortality (RR 0.96, 95% CI 0.68-1.36) were comparable between groups. These findings suggest DOACs are an effective alternative to LMWHs, though careful selection is warranted for high-bleeding-risk patients [21]. When looking at individual major trials, the SELECT-D trial demonstrated that rivaroxaban significantly reduced the six-month VTE recurrence rate to 4% compared to 11% with dalteparin (HR 0.43, 95% CI 0.19-0.99) [17]. Similarly, the Hokusai VTE Cancer trial showed that edoxaban was noninferior to dalteparin, with a primary event rate of 12.8% versus 13.5%, respectively, supporting its role as an alternative [22]. In the Caravaggio trial, apixaban showed comparable outcomes to dalteparin in terms of VTE recurrence (5.6% vs. 7.9%; HR 0.63, 95% CI 0.37-1.07; p < 0.001), with no significant increase in bleeding risk [16]. Additional trials comparing DOACs to warfarin in broader VTE populations offer further insights: the RE-COVER study found dabigatran as effective as warfarin [23], EINSTEIN PE/DVT confirmed rivaroxaban's noninferiority [24], AMPLIFY showed apixaban was noninferior in VTE/VTE-related death [24], and Hokusai-VTE found edoxaban noninferior for recurrent DVT/PE [22]. These trials collectively support the use of DOACs as effective and often safer alternatives to traditional therapies in both cancer and non-cancer-associated VTE contexts.

A summary of the major clinical trials is presented in Table 2.

**Table 2:** Major clinical trials in CAT assessing anticoagulation RCT: randomized controlled trial, VTE: venous thromboembolism, DOAC: direct oral anticoagulant, CAT: cancer-associated thrombosis, DVT: deep vein thrombosis.

Study	Design	Result	Conclusion
SELECT-D	RCT: rivaroxaban vs. dalteparin	Six-month VTE recurrence: 11% (dalteparin), 4% (rivaroxaban) (HR 0.43, 95% CI 0.19-0.99)	Rivaroxaban had lower VTE recurrence vs. dalteparin
Hokusai VTE Cancer	RCT: edoxaban vs. dalteparin	Primary event in 12.8% (edoxaban) vs. 13.5% (dalteparin) (HR 0.97, 95% CI 0.70-1.36; p = 0.006)	Edoxaban noninferior to dalteparin for recurrent VTE
Caravaggio	RCT: DOAC (apixaban) vs. LMWH (dalteparin)	VTE in 5.6% (apixaban) vs. 7.9% (dalteparin) (HR 0.63, 95% CI 0.37-1.07; p < 0.001)	Apixaban noninferior to dalteparin for CAT
RE-COVER	Dabigatran vs. warfarin	Symptomatic recurrent VTE or VTE-related death. The difference in risk was 0.4 percentage points (95% CI −0.8 to 1.5; HR 1.10; 95% CI 0.65-1.84)	A fixed dose of dabigatran is as effective as warfarin
EINSTEIN PE/DVT	Rivaroxaban vs. enoxaparin plus a vitamin K antagonist	Recurrent VTE (HR 0.68, 95% CI 0.44-1.04, p < 0.001)	Rivaroxaban noninferior to warfarin for VTE
AMPLIFY	Apixaban vs. subcutaneous enoxaparin, followed by warfarin	VTE/VTE-related death (RR 0.84, 95% CI 0.60-1.18)	Apixaban noninferior to warfarin
Hokusai-VTE	Edoxaban vs. warfarin	Recurrent VTE (HR 0.89, 95% CI 0.70-1.13, p < 0.001)	Edoxaban noninferior to warfarin in recurrent DVT/PE

Metabolism, dosing, monitoring, and duration of therapy

Dabigatran is administered as the prodrug dabigatran etexilate, which is rapidly hydrolyzed to its active form by plasma esterases. While food intake delays its absorption, it does not affect overall bioavailability. Dabigatran is primarily eliminated via the kidneys, and dose adjustment is necessary in patients with renal impairment. Its use is generally not recommended in individuals with an estimated glomerular filtration rate below 30 mL/min [25]. Apixaban is absorbed in the gastrointestinal tract, and its bioavailability is unaffected by food. It is primarily metabolized by cytochrome P450 3A4 (CYP3A4), with approximately 25% of the administered dose excreted as metabolites in urine and feces [26]. Rivaroxaban is rapidly absorbed, achieving peak plasma concentrations within 2-4 hours after administration. Its oral bioavailability is high (80%-100%) for the 10-mg dose regardless of food, but the 15-mg and 20-mg tablets require food for optimal absorption [27]. A comparative summary of these factors is presented in Table 3.

**Table 3:** Pharmacokinetics and dosing considerations for direct oral anticoagulants (DOACs) used in cancer-associated thrombosis between different DOAC subtypes VTE: venous thromboembolism.

DOACs	Absorption	Metabolism	Dose adjustment	Special notes
Apixaban	Intestinal absorption: food does not affect bioavailability	Metabolized by CYP3A4; renal and hepatobiliary elimination	Not required in mild-to-moderate renal impairment; caution in severe renal impairment	Preferred for initial treatment and short-/long-term use in active cancer
Rivaroxaban	Rapid absorption; food increases bioavailability and plasma concentration	Metabolized in the liver by CYP450, CYP3A4, and CYP2J2; renal and hepatobiliary elimination	Required in renal and hepatic impairment	Preferred for short-term and long-term VTE treatments; food intake improves
Edoxaban	Absorbed orally; no significant impact by food	Metabolized minimally; elimination via kidney and hepatobiliary route	Required in renal impairment and weight-based dosing	Effective in VTE and active cancer treatment; needs renal monitoring
Dabigatran	Prodrug form; food delays absorption but does not affect bioavailability	Hydrolyzed to active form by plasma esterases; renal elimination	Required in renal impairment; contraindicated in renal failure	Direct thrombin inhibitor; selective for thrombus-bound and free thrombin

Current guidelines outline specific dosing strategies for each DOAC used in CAT. Rivaroxaban is typically prescribed at 15 mg twice daily for the first 21 days, followed by 20 mg once daily. Apixaban is initiated at 10 mg twice daily for seven days, then reduced to 5 mg twice daily [28]. Dabigatran requires at least five days of initial parenteral anticoagulation with LMWH before starting 150 mg twice daily [29]. DOACs do not require routine coagulation monitoring, which is a key advantage over VKAs. However, periodic evaluation of renal and hepatic function is advised, particularly in patients with comorbid conditions, advanced malignancies, or those receiving nephrotoxic or hepatotoxic therapies.

The recommended duration of anticoagulation is around six months, with continuation beyond that based on factors such as cancer progression, thrombotic risk, and bleeding risk. While multiple studies have established the efficacy and safety of DOACs in cancer patients, concerns remain regarding their use in certain populations, particularly those with gastrointestinal or genitourinary malignancies, where agents like rivaroxaban and edoxaban have been associated with increased bleeding risk [30]. Consequently, the selection of anticoagulant therapy must be individualized, taking into account the cancer type and location, performance status, renal and hepatic function, platelet count, and potential drug-drug interactions, especially with chemotherapy or targeted therapies. Patients with advanced cancers or those receiving intensive chemotherapy may need closer monitoring and frequent reassessment of anticoagulation safety and efficacy. Additionally, biomarkers such as D-dimer levels, platelet counts, and evolving imaging or clinical indicators can help refine thrombotic risk stratification and tailor the intensity or duration of therapy. Although DOACs simplify management through oral administration and fewer monitoring requirements, ongoing clinical assessment and patient-specific adjustments remain essential. In this complex and high-risk population, balancing the benefits of DOACs with their potential risks requires an individualized and adaptive approach to optimize outcomes [31,32].

Patient-centered approach

**Figure 3:**
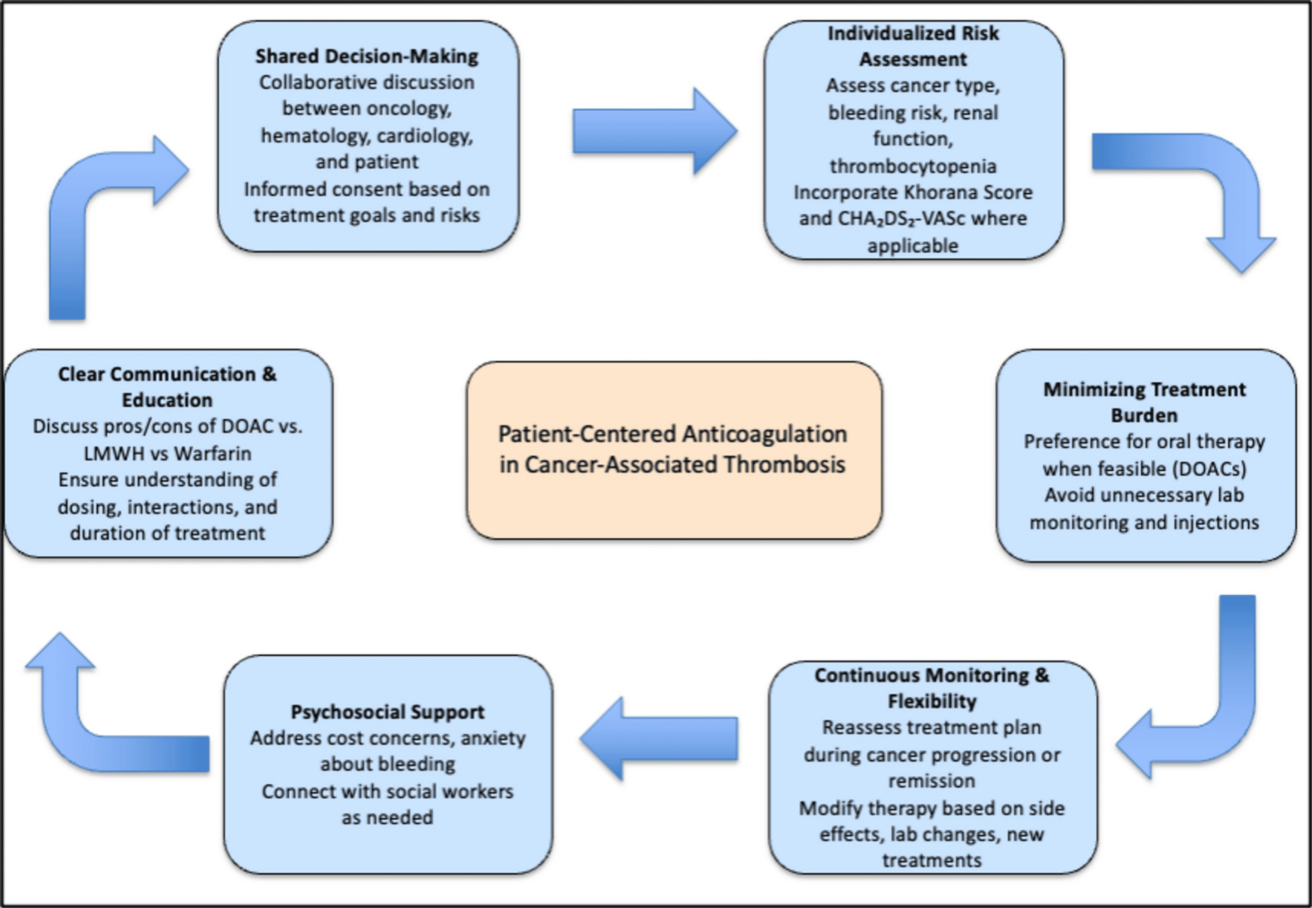
Patient-centered approach in anticoagulation of patients for cancer-associated thrombosis Image Credit: Authors' original creation. This image was created using PowerPoint (Microsoft Corp., Redmond, WA, US).

In managing CAT, a patient-centered approach to anticoagulation has become increasingly vital, reflecting a shift from generalized guidelines toward individualized, holistic care. Patients with cancer represent a highly heterogeneous population, with variable thrombotic and bleeding risks, comorbidities, treatment goals, and psychosocial needs. Integrating patient preferences and clinical complexity into anticoagulant decision-making is essential. The first cornerstone of patient-centered care is shared decision-making [33], where patients are actively involved in selecting anticoagulation strategies that align with their values, preferences, and overall treatment goals. For instance, patients undergoing active chemotherapy may prefer oral agents to avoid the burden of injections, whereas others with gastrointestinal cancers may opt for LMWH due to bleeding concerns [34]. Effective shared decision-making often involves collaboration among oncologists, hematologists, cardiologists, and primary care providers, especially in patients with competing risks such as cardiovascular disease. The second pillar is individualized risk assessment, which involves evaluating each patient’s cancer type, stage, platelet count, renal and hepatic function, and bleeding history. Tools such as the Khorana Score help identify patients at higher risk of thrombosis, while bleeding risk calculators and clinical judgment guide therapy adjustments [35]. It is important to take into account the prothrombotic environment in malignancy, noting how tumor biology, treatment (e.g., chemotherapy or angiogenesis inhibitors), and patient-related factors (e.g., immobilization, surgery) contribute to dynamic thrombotic risk profiles. Additionally, real-world considerations such as renal insufficiency or thrombocytopenia, common in patients with hematologic or advanced cancers, require careful DOAC selection or dose adjustment [36]. Another central aspect of a patient-centered approach is clear communication and patient education. Many patients face confusion or fear surrounding anticoagulant therapy, particularly regarding the duration of treatment, drug interactions, and the risk of recurrence or bleeding. Providers must ensure that patients understand the rationale behind anticoagulation, the comparative safety of DOACs versus LMWH, and how cancer status or treatment changes may impact anticoagulation strategy. Education is also important with regard to identifying bleeding signs, medication adherence, and how to manage therapy interruptions around procedures. Furthermore, the choice of anticoagulant must take into account health literacy, caregiver involvement, and language barriers [37]. The fourth component is minimizing treatment burden, which addresses both the physical and psychological toll of prolonged anticoagulation and anti-cancer drugs’ side effects. Oral agents such as apixaban and rivaroxaban offer a more convenient alternative to LMWH, especially for long-term therapy in the outpatient setting. They eliminate the discomfort of daily injections and reduce the need for laboratory monitoring, thereby improving adherence [37]. Additionally, several classes of anticancer therapies are linked to specific types of cardiac toxicity, which is important to consider when managing patients receiving DOACs. Anthracyclines, such as doxorubicin, can cause direct cardiotoxicity. Anti-metabolites may lead to ischemia, while interleukins are associated with inflammation that can affect the heart. Bruton’s tyrosine kinase inhibitors can interfere with kinase signaling, contributing to arrhythmias. Immune checkpoint inhibitors are associated with immune-related cardiac effects like myocarditis, pericarditis, and vasculitis. Anti-VEGF therapies and BCR-ABL TKIs may contribute to endothelial damage and, in some cases, thrombotic microangiopathy [38]. Equally important is psychosocial support, a domain often underemphasized in anticoagulation discussions. Cancer patients frequently experience anxiety related to both their malignancy and the risk of thromboembolism or bleeding. Financial needs, logistical barriers to care, and fear of medication side effects can all undermine adherence or increase distress. Engaging social workers, patient navigators, and support groups can mitigate these challenges. Additionally, clinicians should proactively address patient concerns about prognosis, end-of-life planning, or transitions in care, especially when anticoagulation is initiated in advanced cancer stages. Lastly, the principle of continuous monitoring and flexibility ensures that anticoagulation management adapts to the patient’s evolving clinical course. Cancer progression, remission, treatment response, or new procedures may necessitate anticoagulant changes [37]. For example, a patient initially started on dabigatran for CAT may need a temporary interruption for surgery or dose reduction due to renal decline. Ongoing follow-up and reassessment are essential to optimize outcomes and reduce complications. Clinicians should monitor renal and hepatic function, platelet counts, and assess for any new drug interactions, especially with cytotoxic or targeted therapies. The use of a multidisciplinary team, routine check-ins, and electronic health record alerts can support this adaptive care model. Adopting a patient-centered approach to anticoagulation in cancer-associated thrombosis requires moving beyond one-size-fits-all recommendations [37]. Such an approach not only improves adherence and safety but also empowers patients in managing their complex care journey. As evidence grows for the efficacy and safety of DOACs in select cancer populations, aligning anticoagulation strategies with patient values and clinical realities will be essential for optimizing outcomes in oncology practice (Figure 3).

## Conclusions

DOACs have become a viable alternative to LMWH for the treatment of CAT, offering similar efficacy with greater convenience, improved adherence, and minimal need for monitoring. Current guidelines support their use in select cancer patients, particularly those without high bleeding risk or significant renal/hepatic impairment. However, caution is warranted in specific patient populations such as patients with GI/GU malignancies or thrombocytopenia. Real-world data and long-term safety studies are still needed, especially in complex oncology populations. Ultimately, DOAC use in CAT should be individualized, balancing thrombotic and bleeding risks through a multidisciplinary, patient-centered approach.
